# Treatment times in breast cancer patients receiving neoadjuvant vs adjuvant chemotherapy: Is efficiency a benefit of preoperative chemotherapy?

**DOI:** 10.1002/cam4.2912

**Published:** 2020-02-25

**Authors:** Nicole M. Melchior, Darren B. Sachs, Gabrielle Gauvin, Cecilia Chang, Chihsiung E. Wang, Elin R. Sigurdson, John M. Daly, Allison A. Aggon, Shelly B. Hayes, Elias I. Obeid, Richard J. Bleicher

**Affiliations:** ^1^ Department of Surgical Oncology Fox Chase Cancer Center Philadelphia PA USA; ^2^ Biostatistics Core NorthShore University HealthSystem Research Institute Evanston IL USA; ^3^ Department of Radiation Oncology Fox Chase Cancer Center Philadelphia PA USA; ^4^ Department of Medical Oncology Fox Chase Cancer Center Philadelphia PA USA

**Keywords:** breast cancer, cancer management, neoadjuvant chemotherapy, surgery

## Abstract

**Background/Objective:**

Delays in times to surgery, chemotherapy, and radiotherapy impair survival in breast cancer patients. Neoadjuvant chemotherapy (NAC) confers equivalent survival to adjuvant chemotherapy (AC), but it remains unknown which approach facilitates faster initiation and completion of treatment.

**Methods:**

Women ≥18 years old with nonrecurrent, noninflammatory, clinical stage I‐III breast cancer diagnosed between 2004 and 2015 who underwent both surgery and chemotherapy were reviewed from the National Cancer Database.

**Results:**

Among 155 606 women overall, 28 241 patients received NAC and 127 365 patients received AC. NAC patients had higher clinical T and N stages (35.8% T3/4 vs 4.9% T3/4; 14.4% N2/3 vs 3.7% N2/3). After adjusting for stage and other factors, NAC patients had longer times to begin treatment (36.1 vs 35.4 days adjusted, *P* = .15), and took significantly longer to start radiotherapy (240.8 vs 218.2 days adjusted, *P* < .0001), and endocrine therapy (301.6 vs 275.7 days adjusted, *P* < .0001). Unplanned readmissions (1.2% vs 1.7%), 30‐day mortality (0.04% vs 0.01%), and 90‐day mortality (0.30% vs 0.08%) were all low and clinically insignificant between NAC and AC.

**Conclusion:**

Compared to patients receiving AC, those receiving NAC do not start treatment sooner. In addition, patients receiving NAC do not complete treatment faster. Although there are clear indications for administering NAC vs AC, rapidity of treatment should not be considered a benefit of giving chemotherapy preoperatively.

## Graphical abstract

This study evaluated the time to initiation and completion of treatment in breast cancer patients undergoing neoadjuvant chemotherapy (NAC) vs adjuvant chemotherapy. We found that NAC did not expedite the start or completion of treatment.

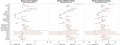

## 1 INTRODUCTION

Multiple prospective randomized trials^1^, ^2^, ^3^ have demonstrated no differences in overall survival (OS) or improved disease free survival (DFS) in patients receiving neoadjuvant chemotherapy (NAC) vs adjuvant chemotherapy (AC) for the treatment of breast cancer. NAC is being used more commonly, especially in cases of triple negative disease and human epidermal growth factor receptor 2 (HER2) positive disease^4^, ^5^ due to the dramatic clinical and pathologic responses often seen. Pathologic complete response rates for triple negative disease range from 23.2% to 33.6%, and for HER2 positive, hormone‐receptor negative disease from 38.7% to 66.2%.^6^, ^7^

NAC is also appropriate for many breast cancer patients with large primary tumors who desire breast conservation,^1^, ^8^, ^9^ and its use may also downstage the axilla before nodal evaluation.^1^, ^8^, ^10^ It can also be used with the goal of eliminating systemic micrometastatic disease,^11^ and to safely delay surgery in certain situations; for example, by allowing time to medically optimize a patient prior to surgery or providing an opportunity to have genetic testing performed.^9^

Additionally, times to treatment have become important, and longer times to surgery, chemotherapy, and radiotherapy all confer modest but significant impairments in survival.^12^ While we were unable to find evidence in the published medical literature, it is thought by some that NAC is typically initiated more quickly than proceeding directly to surgery. Despite this view, there is no data available, to our knowledge, regarding the time it takes patients undergoing NAC to start chemotherapy, as vs the time it takes patients to have surgery first. This study was therefore performed to evaluate the times to start and complete breast cancer treatment in each of these settings to assess whether rapidity of treatment is a benefit of NAC.

## 2 MATERIALS AND METHODS

**Figure 1:**
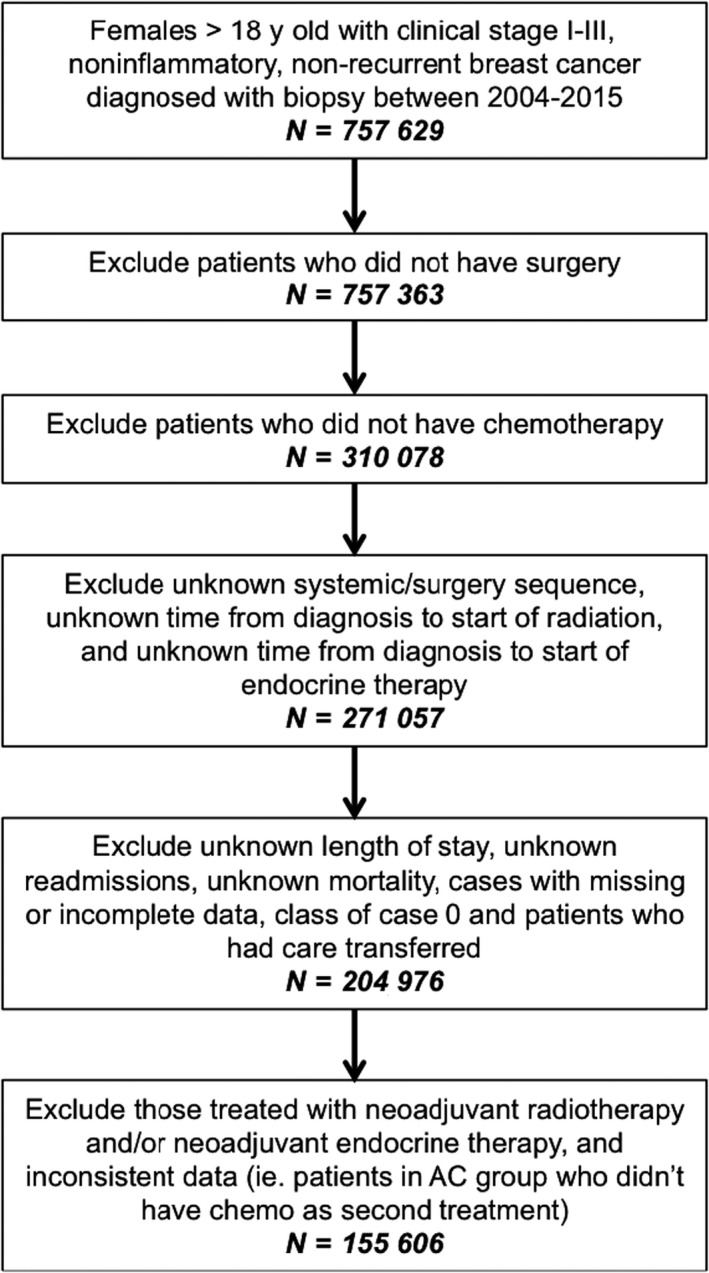
Exclusion diagram

Data were obtained from the National Cancer Database (NCDB). Women ≥18 years old with newly diagnosed, nonrecurrent, noninflammatory, clinical stage I‐III breast cancer diagnosed between 2004 and 2015 whose treatment included both surgery and chemotherapy were selected. Patients with recurrent disease, multiple tumors and/or inflammatory breast cancer were also excluded due to their bad prognosis and varied treatment paradigms, and patients in whom a biopsy was not documented in the dataset were excluded. Remaining exclusions are elaborated in Figure 1.

**Figure 2:**
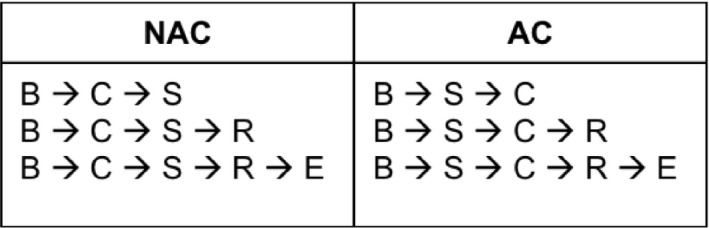
Characterization of patients based on treatment sequence. Patients were initially divided into 2 categories, based on whether they were treated with neoadjuvant chemotherapy or adjuvant chemotherapy. Within those groups, they were then further characterized into 3 groups: Those treated with only surgery and chemotherapy, those who also had radiation, and those who also had radiation and endocrine therapy. These individual groups represent the cohorts included in this study. Times to start treatment (time from biopsy to surgery, or from biopsy to chemotherapy) included all groups. Times to radiation included only those having that modality. Times to complete treatment were measured in those having endocrine therapy. Abbreviations: AC, Adjuvant chemotheraphy; B, Time of biopsy; C, Chemotherapy; E, Endocrine theraphy; NAC, Neoadjuvant chemotheraphy; R, Radiotheraphy; S, Surgery.

Patients were then divided into two groups; those who received NAC and those who received AC. They were then further stratified based on whether they also received radiotherapy and endocrine therapy (Figure 2). Treatment times were measured from time of biopsy to start of first treatment (with first treatment being chemotherapy for NAC group, and surgery for AC group). Time from biopsy to the start of radiotherapy was also measured for all patients who received radiation. Time from biopsy to the start of endocrine therapy was also analyzed. The start of endocrine therapy was used as a surrogate for the end of treatment. Thus, only patients who received endocrine therapy (n = 52 264) were included in that portion of the analysis.

Comparisons between the groups were performed using Student's *t* test for continuous variables and chi‐squared test for categorical variables. To minimize the potential selection bias, treatment times were adjusted for facility volume, age, race, education, insurance, income, setting (urban vs rural vs metropolitan), facility distance, treatment at more than one facility, Charlson comorbidity index, histology, grade, clinical T stage, clinical N stage, AJCC clinical stage, pathologic T stage, pathologic N stage, and phenotype (estrogen, progesterone and HER2) by propensity score with 1:1 matching method. Multiple regression models with log‐transformed treatment times (due to skewed distribution) and with length of hospital stay (approximately normal distribution), as well as multivariable logistic regression models with readmission and 30‐ and 90‐day mortality were constructed to determine predictors associated with these clinical outcomes.

All analyses were performed using SAS 9.4 (SAS Inc). Statistical tests were two‐sided, and a *P*‐value of <.05 was considered statistically significant.

## 3 RESULTS

### 3.1 Patient demographics & tumor characteristics

After accounting for exclusion criteria, 155 606 women remained. Mean patient age was 54.4 ± 11.3 years. Most patients were white (74.8%). Of these, 28 241 patients received NAC and 127 365 patients received AC. Notably, patients treated with NAC tended to be younger (51.9 ± 11.6 years vs 54.9 ± 11.1 years, *P* < .0001). On presentation, they also had larger tumors (cT3‐cT4: 35.8% vs 4.9%, *P* < .0001) and greater nodal involvement (cN2‐cN3: 14.4% vs 3.7%, *P* < .0001) as compared with those having AC. Most patients had ductal histology (86.47%) and the most common tumor phenotype was hormone receptor positive and HER2 negative (55.98%) (Table 1).

**Table 1:** Cohort characteristics

	Overall (N = 155, 606)		NAC (N = 28 241)		AC (N = 127 365)		*P*
	N	%	N	%	N	%	
Facility characteristics							
Facility volume							
Low (0‐50 cases/y)	117 716	75.65	20 908	74.03	96 808	76.01	<.0001
Mid (51‐100 cases/y)	28 945	18.60	5988	21.20	22 957	18.02	
High (>100 cases/y)	8945	5.75	1345	4.76	7600	5.97	
Patient characteristics							
Age, mean ± SD	54.36 ± 11.28		51.93 ± 11.58		54.90 ± 11.14		<.0001
Race							
Caucasian	116 400	74.80	18 821	66.64	97 579	76.61	<.0001
African American	21 398	13.75	5319	18.83	16 079	12.62	
Hispanic	9671	6.22	2448	8.67	7223	5.67	
Asian‐Pacific Islander	5688	3.66	1141	4.04	4547	3.57	
Other/unknown	2449	1.57	512	1.81	1937	1.52	
Education							
21% or more	23 558	15.20	5141	18.29	18 417	14.51	<.0001
13%‐20.9%	37 575	24.20	7146	25.43	30 369	23.93	
7%‐12.9%	50 883	32.83	8856	31.51	42 027	33.12	
<7%	43 042	27.77	6960	24.77	36 082	28.43	
Insurance							
Not Insured	4530	2.91	1405	4.98	3125	2.45	<.0001
Private insurance	102 627	65.95	17 907	63.41	84 720	66.52	
Medicaid	13 916	8.94	3760	13.31	10 156	7.97	
Medicare	31 102	19.99	4472	15.84	26 630	20.91	
Other Government	1751	1.13	324	1.15	1427	1.12	
Unknown	1680	1.08	373	1.32	1307	1.03	
Income							
Less than $38 000	24 203	15.62	4984	17.74	19 219	15.15	<.0001
$38 000‐$47 999	33 145	21.39	6141	21.86	27 004	21.29	
$48 000‐$62 999	41 486	26.77	7536	26.82	33 950	26.76	
$63 000+	56 112	36.21	9436	33.58	46 676	36.80	
Urban/rural							
Metro	130 651	86.11	24 127	87.73	106 524	85.75	<.0001
Urban	18 708	12.33	3017	10.97	15 691	12.63	
Rural	2372	1.56	356	1.29	2016	1.62	
Facility distance (miles)							
<25	127 811	82.47	22 897	81.51	104 914	82.69	<.0001
25‐50	17 061	11.01	3034	10.80	14 027	11.06	
50‐75	4793	3.09	927	3.30	3866	3.05	
>75	5306	3.42	1232	4.39	4074	3.21	
Treated at more than one facility							
No	123 765	79.54	21 834	77.31	101 931	80.03	<.0001
Yes	31 841	20.46	6407	22.69	25 434	19.97	
Charlson Comorbidity Index							
0	133 123	85.55	24 852	88.00	108 271	85.01	<.0001
1	19 001	12.21	2885	10.22	16 116	12.65	
2	2847	1.83	403	1.43	2444	1.92	
3	635	0.41	101	0.36	534	0.42	
Tumor characteristics							
Histology							
Ductal	134 553	86.47	24 627	87.20	109 926	86.31	<.0001
Lobular	11 062	7.11	1772	6.27	9290	7.29	
Other/unknown	9991	6.42	1842	6.52	8149	6.40	
Grade							
Grade 1	12 672	8.14	1564	5.54	11 308	8.72	<.0001
Grade 2	55 677	35.78	9121	32.30	46 556	36.55	
Grade 3 & anaplastic	80 522	51.75	15 539	55.02	64 983	51.02	
Unknown	6735	4.33	2017	7.14	4718	3.70	
Clinical T stage							
0	122	0.08	40	0.14	82	0.06	<.0001
1	73 290	47.10	3926	13.90	69 364	54.46	
2	63 701	40.94	13 839	49.00	49 862	39.15	
3	12 797	8.22	7251	25.68	5546	4.35	
4	3574	2.30	2869	10.16	705	0.55	
Other/unknown	2122	1.36	316	1.12	1806	1.42	
Clinical N stage							
0	111 546	71.68	11 475	40.63	100 071	78.57	<.0001
1	30 930	19.88	12 041	42.64	18 889	14.83	
2	6044	3.88	2689	9.52	3355	2.63	
3	2737	1.76	1363	4.83	1374	1.08	
Other/unknown	4349	2.79	673	2.38	3676	2.89	
AJCC clinical stage							
I	64 258	41.30	2106	7.46	62 152	48.80	<.0001
II	74 083	47.61	16 268	57.61	57 815	45.39	
III	17 263	11.09	9866	34.94	7397	5.81	
Pathologic T stage							
0	1345	0.86	1292	4.57	53	0.04	<.0001
1	65 876	42.34	11 604	41.09	54 272	42.61	
2	57 623	37.03	8040	28.47	49 583	38.93	
3	9965	6.40	3057	10.82	6908	5.42	
4	1786	1.15	997	3.53	789	0.62	
Other/unknown	19 011	12.22	3251	11.51	15 760	12.37	
Pathologic N stage							
0	69 078	46.14	11 463	41.93	57 615	47.08	<.0001
1	40 437	27.01	7457	27.28	32 980	26.95	
2	14 399	9.62	3724	13.62	10 675	8.72	
3	6742	4.50	1694	6.20	5048	4.12	
Other/unknown	19 068	12.74	3000	10.97	16 068	13.13	
Molecular marker status							
HER2+, HR+	11 676	11.20	2149	10.96	9527	11.25	<.0001
HER2+, HR−	7948	7.62	1927	9.82	6021	7.11	
HER2−, HR+	58 377	55.98	9185	46.83	49 192	58.10	
HER2−, HR−	26 274	25.20	6353	32.39	19 921	23.53	
Regional lymph node status							
Negative	78 037	52.97	12 230	47.55	65 807	54.11	<.0001
Positive	69 290	47.03	13 489	52.45	55 801	45.89	

Abbreviations: AC, adjuvant chemotherapy; AJCC, American Joint Committee on Cancer; HER2, human epidermal growth factor receptor 2; HR, hormone receptor; NAC, neoadjuvant chemotherapy.

### 3.2 Times to treatment

Unadjusted time comparisons were first determined (Table 2). Time from biopsy to first treatment was 35.6 ± 27.5 days in patients treated with NAC, vs 33.4 ± 22.9 days in patients treated with AC (*P* < .0001). Unadjusted time from biopsy to radiation was 243.2 ± 58.8 days in NAC vs 208.7 ± 54.6 days in AC (*P* < .0001), and unadjusted time from biopsy to start of endocrine therapy was 305.4 ± 77.6 days in NAC vs 268.3 ± 71.1 days in AC (*P* < .0001). After propensity score matching, adjusted times are detailed in Table 2. NAC patients had a similar time to begin treatment compared to AC patients, but took significantly longer to start radiotherapy and endocrine therapy.

**Table 2:** Unmatched and propensity score‐matched time comparisons

	NAC mean (d)	AC mean (d)	*P*‐value	Δ
Unmatched				
Biopsy to first treatment	35.6 ± 27.5	33.4 ± 22.9	<.0001	2.2
Biopsy to radiation	243.2 ± 58.8	208.7 ± 54.6	<.0001	34.5
Biopsy to endocrine therapy	305.4 ± 77.6	268.3 ± 71.1	<.0001	37.1
Matched				
Biopsy to first treatment	36.1 ± 30.8	35.4 ± 25.7	.15	0.7
Biopsy to radiation	240.8 ± 59.2	218.2 ± 56.6	<.0001	22.6
Biopsy to endocrine therapy	301.6 ± 70.4	275.7 ± 66.5	<.0001	25.9

Abbreviations: AC, adjuvant chemotherapy; NAC, neoadjuvant chemotherapy.

### 3.3 Factors that influence treatment times

**Figure 3:**
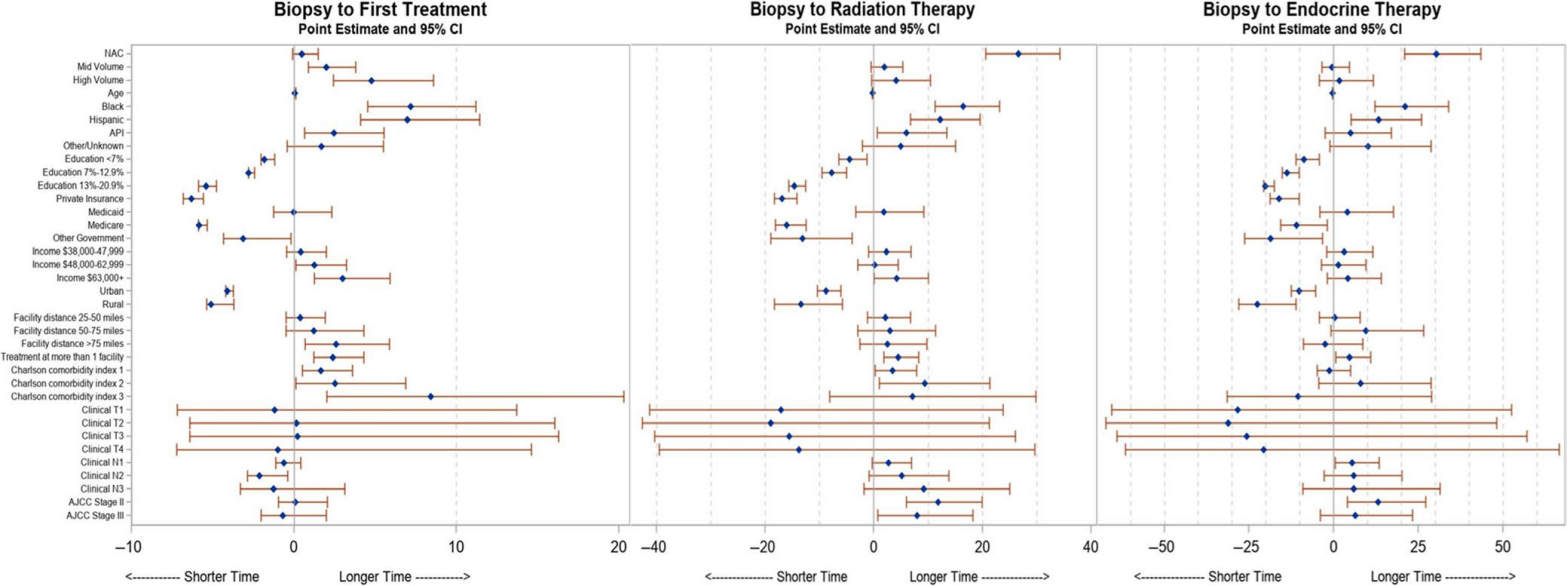
Forest plots demonstrating factors that influence treatment times

As depicted in Figure 3, predictors of times to treatment initiation were examined. The factors associated with a longer time to treatment initiation included higher volume facilities, black and Hispanic patients, increased income, and increased Charlson comorbidity index. Patients treated at more than one facility, along with black and Hispanic patients were more likely to have longer times to treatment initiation as well as delays in starting radiation and endocrine therapy. Factors associated with shorter times to first treatment, radiation therapy, and endocrine therapy included higher education level, private insurance, and treatment in an urban or rural setting (as opposed to a metropolitan setting).

### 3.4 Outcomes

After propensity score matching, there was no difference in the length of hospital stay among patients treated with NAC vs AC (1.16 ± 4.93 vs 1.28 ± 5.90 days, *P* = .14). Rates of unplanned readmission were slightly lower in patients treated with NAC vs AC (1.18% vs 1.72%), however this was not clinically significant. Similarly, 30‐day mortality rates (0.04% vs 0.01%) and 90‐day mortality rates (0.30% vs 0.08%) were also not clinically different since the rates were so low.

### 3.5 Independent predictors of increased length of hospital stay, readmission & mortality

Multivariable analyses were performed to determine independent predictors of increased length of hospital stay, readmission, and 30‐ and 90‐day mortality rates. Predictors of increased length of hospital stay were Charlson comorbidity score of 3 (0.36 days longer, 95% confidence interval [CI] 0.01‐1.19, *P* = .04), Charlson comorbidity score of 2 (0.26 days longer, 95% CI 0.06‐0.66, *P* < .05), higher income (0.15 days longer, 95% CI 0.04‐0.37, *P* < .05), black race (0.13 days longer, 95% CI 0.05‐0.29, *P* < .0001) and Hispanic ethnicity (0.11 days longer, 95% CI 0.20‐0.30, *P* = .01). Charlson comorbidity score of 2 (odds ratio [OR] 2.3, 95% CI 1.52‐3.33, *P*=<0.0001) was found to be the only predictor of increased readmission rates. Predictors of increased 30‐day mortality included Charlson comorbidity score of 3 (OR 15.46, 95% CI 1.55‐153.79, *P* = .02), patients with HER2 positive, hormone receptor negative disease (OR 6.75, 95% CI 1.05‐43.37, *P* = .04), higher income (OR 6.29, 95% CI 1.15‐34.49, *P* = .03), and black race (OR 3.70, 95% CI 1.40‐9.78, *P*=.01). Predictors of increased 90‐day mortality included Charlson comorbidity score of 2 (OR 3.18, 95% CI 1.15‐8.83, *P* = .03), higher income (OR 3.08, 95% CI 1.06‐8.95, *P* = .04), facility distance of 25‐50 miles (OR 2.5, 95% CI 1.17‐5.37, *P* = .02), black race (OR 1.89, 95% CI 1.01‐3.53, *P* = .05), and older age (1.06, 95% CI 1.03‐3.53, *P* = .05) (Table 3).

**Table 3:** Independent predictors of increased length of stay, readmission & mortality

	Increased length of stay	Increased readmission	Increased 30‐d mortality	Increased 90‐d mortality
Black race	**√**		**√**	**√**
Hispanic ethnicity	**√**			
Older age				**√**
Higher income			**√**	**√**
Charlson comorbidity score 2	**√**	**√**		
Charlson comorbidity score 3	**√**		**√**	
Facility distance 25‐50 miles				**√**
HER2+, HR− disease			**√**	

Note

Factors that were also evaluated but were not significant were education level, clinical nodal status, tumor histology (ductal vs lobular), HER2−/HR+ tumors and triple negative tumors.

Abbreviations: HER2, human epidermal growth factor receptor 2; HR, hormone receptor.

## 4 DISCUSSION

NAC has benefits in the management of breast cancer including shrinking the size of the primary tumor to allow for increased rates of breast conservation, and downstaging disease in the axilla. Some proponents of NAC also believe that it shortens the time to treatment initiation compared to upfront surgery, however, to our knowledge, there is no data substantiating this claim, even though the published medical literature does provide data relating to general delays in breast cancer care.^13^, ^14^

The interest in whether NAC improves time to treatment initiation likely originates from data showing that a longer time to surgery is associated with lower OS and DFS in breast cancer patients, with the largest decline seen in patients having stage I and II disease.^13^ While delays to surgery are significant, a delay of >90 days as vs ≤30 days has been found to be associated with only a 3%‐5% decrement in survival.^13^ Similarly, a delay of more than 90 days to initiation of chemotherapy is also associated with worse OS and breast cancer‐specific survival,^15^, ^16^ and a study evaluating patients with hormone receptor‐negative breast cancer found that delaying chemotherapy by 6 weeks resulted in worse OS.^17^ Regarding delays to initiation of adjuvant radiotherapy, the data is mixed, with some studies suggesting that outcomes are unaffected when radiation is given between 8 and 20 weeks postoperatively in patients who do not receive chemotherapy, and within 365 days in patients who do receive chemotherapy.^12^

For these reasons, quality measures currently specify three recommended time intervals^18^: firstly, that radiotherapy should be given within 365 days of diagnosis in women under the age of 70 who have breast conserving surgery. Secondly, that chemotherapy should be considered or administered within 4 months of diagnosis for women under the age of 70 with AJCC T1c, Stage II, or Stage III hormone‐receptor‐negative breast cancer, and thirdly, that tamoxifen or a third‐generation aromatase inhibitor is considered or administered within 1 year of diagnosis for women with AJCC T1c, Stage II, or Stage III hormone receptor positive breast cancer. Compliance with these measures is tracked by accrediting bodies such as the National Accreditation Program for Breast Centers^18^ because of the importance of appropriately initiating treatment without undue delay. Avoiding treatment delays is therefore important to both optimize outcomes and comply with quality measures.

After adjusting for numerous factors, we found that time from biopsy to treatment initiation (either surgery or chemotherapy) was similar in patients receiving NAC vs AC. Thus, NAC does not expedite time to treatment initiation, even though there may be situations where NAC may be desirable if undue preoperative delays are unavoidable.

Delays to surgery also have an impact on time to chemotherapy and this could affect compliance with the chemotherapy quality measure. Patients who undergo mastectomy with or without reconstruction are at a higher risk of having delays to AC due to longer recovery time and higher rate of postoperative complications.^19^ For patients undergoing larger procedures where postoperative recovery time is predicted to be unusually prolonged, it may be reasonable to treat them with chemotherapy upfront.

It must be remembered that times to treatment are likely eclipsed by the “silent interval” of a tumor, which is defined as the period of tumor growth beginning at inception, up until it can be detected. By definition, we cannot know the length of this period of time, but it is generally thought that these intervals frequently comprise the majority of a tumor's lifespan.^20^ It is because of this that delays to surgery, chemotherapy or radiotherapy likely have much less of an impact than typically surmised.

We found that the time to initiation of radiotherapy was 3.2 weeks longer in the NAC group than in the AC group, however, this delay of 3 weeks in the NAC cohort is unlikely to impact clinical outcomes in most cases. Most published studies have found that delays in radiotherapy of at least 8‐20 weeks are required before a decline in survival or an increase in local recurrence is seen.^12^ However, a study evaluating 581 patients specifically treated with NAC and postoperative radiotherapy found that initiating radiation <8 weeks after surgery was associated with improved disease‐specific and OS.^21^ Ultimately, with multidisciplinary treatment, the impact of longer radiotherapy after changing the order of surgery and chemotherapy remains uncertain. However, the current radiotherapy standard requires that radiation be administered within 1 year of diagnosis,^18^ and so clinicians must, at minimum be cognizant of the fact that a 3‐week delay can mean the difference between compliance and noncompliance in terms of quality measures.

A second objective of our study was to assess the time to completion of treatment, and the start of endocrine therapy was used as a surrogate for this time point. Since endocrine therapy is given as an outpatient medication, compliance is difficult to monitor, and lengths of therapy can vary from 5 to 10 years, the start, rather than the completion, of hormonal therapy was felt to be a reliable estimate of the completion of their overall treatment course. Moreover, we are not aware of any similarly sized dataset that assesses either time to completion of endocrine therapy or the level of compliance. We found that time to completion of treatment was 3.7 weeks longer in the NAC group compared to the AC group. Unfortunately, there is no good data, to our knowledge, regarding delays in endocrine therapy on outcomes. Especially with time spans for hormonal therapy on treatment being 5‐10 years, delays over a matter of weeks likely make little difference. Moreover it must be remembered that the expansion of endocrine therapy from 5 to 10 years was to solely provide a 3% survival benefit,^22^ making any delays seen here likely inconsequential. We therefore do not suggest that the time delay to endocrine therapy, which is our surrogate for completion of treatment, in the NAC group has impaired survival, but instead simply that these data demonstrate that it has not expedited or improved outcomes related to potential delays.

In an effort to provide a potential explanation for differences between the NAC group and those having surgery first, we speculated that NAC might increase the risk of perioperative complications, resulting in increased length of hospital stay after surgery due to wound complications or other chemotherapy‐related side effects. However our analysis showed no difference in length of hospital stay between AC and NAC. This is consistent with prior studies that have found that NAC is not associated with an increase in short‐term complications after mastectomy and implant‐based reconstruction^23^ or autologous tissue reconstruction.^24^

Similarly, we also examined whether the longer time to complete treatment in the NAC group was due to higher readmission rates, but surprisingly, patients treated with NAC actually had lower rates of both unplanned and planned readmission. The reason for this is unclear, but perhaps this could be due to the closer follow up and monitoring of these patients while they were undergoing chemotherapy. With regard to mortality rates, as expected, 30‐ and 90‐day mortality rates were very low and clinically insignificant. We therefore cannot attribute the longer time to completion of treatment to an increase in perioperative complications resulting from NAC. The small differences in timing are therefore more likely due to scheduling factors or even due to a self‐imposed delay to wait for recovery from chemotherapy effects before embarking upon surgery out of concern for wound healing.

We found that factors having the greatest correlation with a longer time to treatment initiation were higher volume facilities, increased income, and increased Charlson comorbidity index. Higher volume facilities may have longer wait times for consultations, resulting in delays to surgery or starting NAC. However, treatment at high volume centers has been shown to be associated with improved OS.^25^ It is unclear why higher income was associated with longer treatment times, but the most likely reason is that these patients have the financial means to obtain multiple opinions at different facilities, which lengthens the time from diagnosis to treatment initiation.^26^ Lastly, increased Charlson comorbidity index was also associated with a longer time to treatment initiation, presumably because these patients require more extensive evaluation and clearance before starting NAC or before proceeding to surgery.

We also found that patients treated at more than one facility, along with black and Hispanic patients had longer times to first treatment, radiation therapy and endocrine therapy, consistent with known disparities and the published medical literature. Stage for stage, black women have been found to have higher breast cancer mortality rates than white women,^27^, ^28^ which could be a result of either differences in access to care, or disparities in how care is delivered. Studies have also demonstrated that time from biopsy to treatment initiation is longer in black women^27^ and blacks and Hispanics have been found to have greater delays to surgery,^14^ all consistent with our findings here.

In contrast to income level, higher education level and having private insurance were associated with a shorter time to treatment initiation, radiotherapy, and endocrine therapy which might be explained, respectively, by knowledge of the disease and the imperative to be treated, and having access to more specialists to facilitate care. Being treated in an urban or rural setting as opposed to a metropolitan setting also resulted in shorter treatment times. Despite the smaller volume and potential shorter wait to see a physician, there are likely certain geographical areas which are lacking breast cancer specialists and have a longer delay. All of these findings indicate that, unfortunately, disparities remain in the neoadjuvant setting as well, and are not dependent on or related to treatment order.

Although this study utilizes a large prospectively collected validated dataset, it is limited by the NCDB’s accuracy, which is dictated by how well the cases are coded into it. Additionally, like all datasets, the NCDB dataset is limited to specific variables, and unknown confounders could exist. Length of chemotherapy treatment, type of chemotherapy administered, and time to completion of endocrine therapy are not collected in this dataset, thus limiting our ability to assess how often truncation of those therapies occurs, and how timing is consequently affected. To overcome this, the time to initiation of endocrine therapy was used as a surrogate for the end of treatment. Our study's strengths include the very large size of the dataset utilized and its applicability to the national population at large, as well as its ability to discern precise intervals of treatment.

## 5 CONCLUSION

While there are clear indications for NAC in the treatment of breast cancer, NAC did not result in patients starting or completing treatment faster than those who received AC. Although one might consider its use if prolonged preoperative or postoperative delays are expected, quicker treatment initiation and completion should not be considered a routine benefit of NAC as vs primary surgery, and so this is not an indication to administer chemotherapy upfront.

## AUTHOR CONTRIBUTIONS

Nicole Melchior: Conceptualization, investigation, formal analysis, writing—original draft, writing—review and editing. Darren Sachs: Conceptualization, investigation, writing—review and editing. Gabrielle Gauvin: Conceptualization, investigation, writing—review and editing. Cecilia Chang: Data curation, formal analysis, software, writing—review and editing. Chihsiung Wang: Data curation, formal analysis, software, writing—review and editing. Elin Sigurdson: Conceptualization, project administration, writing—review and editing. John Daly: Conceptualization, project administration, writing—review and editing. Allison Aggon: Conceptualization, project administration, writing—review and editing. Shelly Hayes: Project administration, writing—review and editing. Elias Obeid: Project administration, writing—review and editing. Richard Bleicher: Conceptualization, funding acquisition, project administration, writing—original draft, writing—review and editing.

## Data Availability

Research data are not shared.

## References

[1] Fisher B , Bryant J , Wolmark N , et al. Effect of preoperative chemotherapy on the outcome of women with operable breast cancer. J Clin Oncol. 1998;16:2672‐2685. 9704717 10.1200/JCO.1998.16.8.2672

[2] Mamounas EP , Anderson SJ , Dignam JJ , et al. Predictors of locoregional recurrence after neoadjuvant chemotherapy: results from combined analysis of National Surgical Adjuvant Breast and Bowel Project B‐18 and B‐27. J Clin Oncol. 2012;30:3960‐3966. 23032615 10.1200/JCO.2011.40.8369 PMC3488269

[3] Bear HD , Anderson S , Smith RE , et al. Sequential preoperative or postoperative docetaxel added to preoperative doxorubicin plus cyclophosphamide for operable breast cancer: National Surgical Adjuvant Breast and Bowel Project Protocol B‐27. J Clin Oncol. 2006;24:2019‐2027. 16606972 10.1200/JCO.2005.04.1665

[4] Bartsch R , Bergen E , Galid A . Current concepts and future directions in neoadjuvant chemotherapy of breast cancer. Memo. 2018;11:199‐203. 30220926 10.1007/s12254-018-0421-1 PMC6132793

[5] Harbeck N , Gluz O . Neoadjuvant therapy for triple negative and HER2‐positive early breast cancer. Breast. 2017;34(suppl 1):S99‐S103. 28666920 10.1016/j.breast.2017.06.038

[6] Schneeweiss A , Chia S , Hickish T , et al. Pertuzumab plus trastuzumab in combination with standard neoadjuvant anthracycline-containing and anthracycline-free chemotherapy regimens in patients with HER2-positive early breast cancer: a randomized phase II cardiac safety study (TRYPHAENA). Ann Oncol. 2013;24:2278‐2284. 23704196 10.1093/annonc/mdt182

[7] Haque W , Verma V , Hatch S , Suzanne Klimberg V , Brian Butler E , Teh BS . Response rates and pathologic complete response by breast cancer molecular subtype following neoadjuvant chemotherapy. Breast Cancer Res Treat. 2018;170:559‐567. 29693228 10.1007/s10549-018-4801-3

[8] Fisher B , Brown A , Mamounas E , et al. Effect of preoperative chemotherapy on local‐regional disease in women with operable breast cancer: findings from National Surgical Adjuvant Breast and Bowel Project B‐18. J Clin Oncol. 1997;15:2483‐2493. 9215816 10.1200/JCO.1997.15.7.2483

[9] Chatterjee A , Erban JK . Neoadjuvant therapy for treatment of breast cancer: the way forward, or simply a convenient option for patients? Gland Surg. 2017;6:119‐124. 28210563 10.21037/gs.2016.08.07 PMC5293643

[10] Bear HD , Anderson S , Brown A , et al. The effect on tumor response of adding sequential preoperative docetaxel to preoperative doxorubicin and cyclophosphamide: preliminary results from National Surgical Adjuvant Breast and Bowel Project Protocol B‐27. J Clin Oncol. 2003;21:4165‐4174. 14559892 10.1200/JCO.2003.12.005

[11] Bonadonna G , Valagussa P , Zucali R , Salvadori B . Primary chemotherapy in surgically resectable breast cancer. CA Cancer J Clin. 1995;45:227‐243. 7600279 10.3322/canjclin.45.4.227

[12] Bleicher RJ . Timing and delays in breast cancer evaluation and treatment. Ann Surg Oncol. 2018;25:2829‐2838. 29968031 10.1245/s10434-018-6615-2 PMC6123282

[13] Bleicher RJ , Ruth K , Sigurdson ER , et al. Time to surgery and breast cancer survival in the United States. JAMA Oncol. 2016;2:330‐339. 26659430 10.1001/jamaoncol.2015.4508 PMC4788555

[14] Bleicher RJ , Ruth K , Sigurdson ER , et al. Preoperative delays in the US Medicare population with breast cancer. J Clin Oncol. 2012;30:4485‐4492. 23169513 10.1200/JCO.2012.41.7972 PMC3518727

[15] Chavez‐MacGregor M , Clarke CA , Lichtensztajn DY , Giordano SH . Delayed initiation of adjuvant chemotherapy among patients with breast cancer. JAMA Oncol. 2016;2:322‐329. 26659132 10.1001/jamaoncol.2015.3856 PMC5920529

[16] Lohrisch C , Paltiel C , Gelmon K , et al. Impact on survival of time from definitive surgery to initiation of adjuvant chemotherapy for early‐stage breast cancer. J Clin Oncol. 2006;24:4888‐4894. 17015884 10.1200/JCO.2005.01.6089

[17] Abdel‐Rahman O . Impact of timeliness of adjuvant chemotherapy and radiotherapy on the outcomes of breast cancer; a pooled analysis of three clinical trials. Breast. 2018;38:175‐180. 29432980 10.1016/j.breast.2018.01.010

[18] National Accreditation Program for Breast Centers Standards Manual. Chicago, IL: American College of Surgeons; 2018.

[19] de Melo GD , Chavez‐MacGregor M . Delays in adjuvant chemotherapy among breast cancer patients: an unintended consequence of breast surgery? Ann Surg Oncol. 2018;25:1786‐1787. 29600346 10.1245/s10434-018-6415-8

[20] Pearlman AW . Breast cancer‐influence of growth rate on prognosis and treatment evaluation: a study based on mastectomy scar recurrences. Cancer. 1976;38(4):1826‐1833. 991096 10.1002/1097-0142(197610)38:4<1826::aid-cncr2820380460>3.0.co;2-l

[21] Silva SB , Pereira AAL , Marta GN , et al. Clinical impact of adjuvant radiation therapy delay after neoadjuvant chemotherapy in locally advanced breast cancer. Breast. 2018;38:39‐44. 29223797 10.1016/j.breast.2017.11.012

[22] Davies C , Pan H , Godwin J , et al. Long‐term effects of continuing adjuvant tamoxifen to 10 years versus stopping at 5 years after diagnosis of oestrogen receptor‐positive breast cancer: ATLAS, a randomised trial. Lancet. 2013;381(9869):805‐816. 23219286 10.1016/S0140-6736(12)61963-1 PMC3596060

[23] Donker M , Hage JJ , Woerdeman LA , Rutgers EJ , Sonke GS , Vrancken Peeters MJ . Surgical complications of skin sparing mastectomy and immediate prosthetic reconstruction after neoadjuvant chemotherapy for invasive breast cancer. Eur J Surg Oncol. 2012;38:25‐30. 21963981 10.1016/j.ejso.2011.09.005

[24] Tanaka S , Hayek G , Jayapratap P , et al. The impact of chemotherapy on complications associated with mastectomy and immediate autologous tissue reconstruction. Am Surg. 2016;82:713‐717. 27657587

[25] Greenup RA , Obeng‐Gyasi S , Thomas S , et al. The effect of hospital volume on breast cancer mortality. Ann Surg. 2018;267:375‐381. 27893532 10.1097/SLA.0000000000002095 PMC5994238

[26] Bleicher RJ , Chang C , Wang CE , et al. Treatment delays from transfers of care and their impact on breast cancer quality measures. Breast Cancer Res Treat. 2019;173:603‐617. 30443881 10.1007/s10549-018-5046-x

[27] Selove R , Kilbourne B , Fadden MK , et al. Time from screening mammography to biopsy and from biopsy to breast cancer treatment among black and white, women Medicare beneficiaries not participating in a health maintenance organization. Womens Health Issues. 2016;26:642‐647. 27773529 10.1016/j.whi.2016.09.003 PMC5116399

[28] Hunt BR , Whitman S , Hurlbert MS . Increasing Black:White disparities in breast cancer mortality in the 50 largest cities in the United States. Cancer Epidemiol. 2014;38:118‐123. 24602836 10.1016/j.canep.2013.09.009

